# Feasibility study of opportunistic osteoporosis screening on chest CT using a multi-feature fusion DCNN model

**DOI:** 10.1007/s11657-024-01455-7

**Published:** 2024-10-17

**Authors:** Jing Pan, Peng-cheng Lin, Shen-chu Gong, Ze Wang, Rui Cao, Yuan Lv, Kun Zhang, Lin Wang

**Affiliations:** 1 Department of Radiology, The Second Affiliated Hospital of Nantong University, Nantong, 226001 Jiangsu China; 2 Department of Radiology, Nanjing Hospital of Chinese Medicine Affiliated to Nanjing University of Chinese Medicine, Nanjing, 210000 Jiangsu China; 3 School of Electrical Engineering, Nantong University, Nantong, 226001 Jiangsu China

**Keywords:** Osteoporosis, Quantitative CT, Deep learning, Bone mineral density, Feature fusion

## Abstract

***Summary*:**

A multi-feature fusion DCNN model for automated evaluation of lumbar vertebrae L1 on chest combined with clinical information and radiomics permits estimation of volumetric bone mineral density for evaluation of osteoporosis.

**Purpose:**

To develop a multi-feature deep learning model based on chest CT, combined with clinical information and radiomics to explore the feasibility in screening for osteoporosis based on estimation of volumetric bone mineral density.

**Methods:**

The chest CT images of 1048 health check subjects were retrospectively collected as the master dataset, and the images of 637 subjects obtained from a different CT scanner were used for the external validation cohort. The subjects were divided into three categories according to the quantitative CT (QCT) examination, namely, normal group, osteopenia group, and osteoporosis group. Firstly, a deep learning–based segmentation model was constructed. Then, classification models were established and selected, and then, an optimal model to build bone density value prediction regression model was chosen.

**Results:**

The DSC value was 0.951 ± 0.030 in the testing dataset and 0.947 ± 0.060 in the external validation cohort. The multi-feature fusion model based on the lumbar 1 vertebra had the best performance in the diagnosis. The area under the curve (AUC) of diagnosing normal, osteopenia, and osteoporosis was 0.992, 0.973, and 0.989. The mean absolute errors (MAEs) of the bone density prediction regression model in the test set and external testing dataset are 8.20 mg/cm^3^ and 9.23 mg/cm^3^, respectively, and the root mean square errors (RMSEs) are 10.25 mg/cm^3^ and 11.91 mg/cm^3^, respectively. The *R*-squared values are 0.942 and 0.923, respectively. The Pearson correlation coefficients are 0.972 and 0.965.

**Conclusion:**

The multi-feature fusion DCNN model based on only the lumbar 1 vertebrae and clinical variables can perform bone density three-classification diagnosis and estimate volumetric bone mineral density. If confirmed in independent populations, this automated opportunistic chest CT evaluation can help clinical screening of large-sample populations to identify subjects at high risk of osteoporotic fracture.

**Supplementary Information:**

The online version contains supplementary material available at 10.1007/s11657-024-01455-7.

## Introduction

Osteoporosis is a metabolic bone disease characterized by systemic or local bone loss and an increased risk of fracture [1]. Given the aging of the large global population, it is considered a major illness. Osteoporosis is estimated to affect 13.5% of men and 29.0% of women aged 50 and over in China [2]. Worldwide, 19.7% of men and 40.4% of women aged 50 and over suffer from osteoporosis and osteopenia, respectively [3]. Early diagnosis and treatment of osteoporosis can effectively slow the development of bone resorption and reduce the risk of fragility fracture and the incidence of osteoporosis-related complications, alleviating the degree of social burden [4]. The SCOOP (screening for prevention of fractures in older women) study of fragility fracture prevention screening in older women in the UK confirmed a significant 33% reduction in hip fracture incidence in the intervention group compared with the control group [5]. Therefore, early screening and monitoring are essential for the timely prevention and treatment of osteoporosis [1].

For the diagnosis and screening of osteoporosis, the measurement of vertebral bone mineral density (BMD) is an important indicator recommended by the World Health Organization [6]. At present, the main clinical methods for BMD measurement include dual-energy X-ray absorptiometry (DXA) and quantitative computed tomography (QCT) [7]. However, early screening of osteoporosis is difficult. Miller [8] investigated that nearly a quarter of women with high-risk factors for osteoporosis have never undergone BMD measurements due to insufficient understanding of fragility fractures, the need for auxiliary hardware equipment to measure BMD, and additional manual operation costs.

Opportunistic osteoporosis screening [9] refers to the measurement of the vertebral BMD to diagnose osteoporosis during CT scans for other indications, such as lung cancer screening, without additional equipment or increased radiation exposure and without significant additional costs [2].

With the continuous development of machine learning, especially radiomics [10–13] and convolutional neural networks (CNNs) [14–16], an increasing number of osteoporosis cases are diagnosed and screened based on CT images. Radiomics features and deep learning may provide complementary information for predicting osteoporosis [17].

Studies have confirmed that combinations of clinical risk factors can also be used to predict osteoporosis, and it is optimal to add bone mass while using clinical risk factors [18, 19]. However, to the best of our knowledge, few studies have integrated clinical information and radiological features when constructing deep learning multi-feature fusion models for the diagnosis of osteoporosis.

The purpose of this study is to analyze the feasibility and effectiveness of a deep learning fusion model based on chest CT images and clinical information to evaluate bone density status and accurately predict bone density values by using QCT results as a reference standard and to verify the external applicability and generalizability of the fusion model by using an external validation cohort to obtain a low-cost deep learning method as an auxiliary means for the “opportunistic” screening of osteoporosis. This model can be used to guide clinical decision.

## Materials and methods

This retrospective study was approved by the Ethics Committee of Nantong First People’s Hospital (No. 2021KT028), who waived the need for informed consent. The study protocol was implemented according to the Good Clinical Practice guidelines defined by the Helsinki Declaration and the International Conference on Harmonization (ICH).

### Study design and patient population

In this study, the images from two CT scanners (Ingenuity Core 128 CT (Philips Health Care, Holland) and SOMATOM Force CT (Siemens Health Care, Erlangen, Germany)) used for annual health check were collected to form an internal dataset (Ingenuity Core 128 CT) and external validation cohort (SOMATOM Force CT). A total of 1913 subjects who underwent annual health check in our hospital from January 2021 to October 2021 were consecutively collected as the internal dataset. The external validation cohort included 702 subjects who underwent annual health check from February 2022 to October 2022. The inclusion criteria were as follows: (1) age 18 years or older and (2) tube voltage 120 kVp [20] and availability of QCT bone density measurements. The exclusion criteria were as follows: (1) the scanning range did not cover the entire lumbar 2 vertebral body; (2) lumbar 1 and lumbar 2 vertebral fractures, tumors, implants, or previous metal fixation, vertebroplasty; and (3) severe degenerative changes or deformity.

**Fig. 1:**
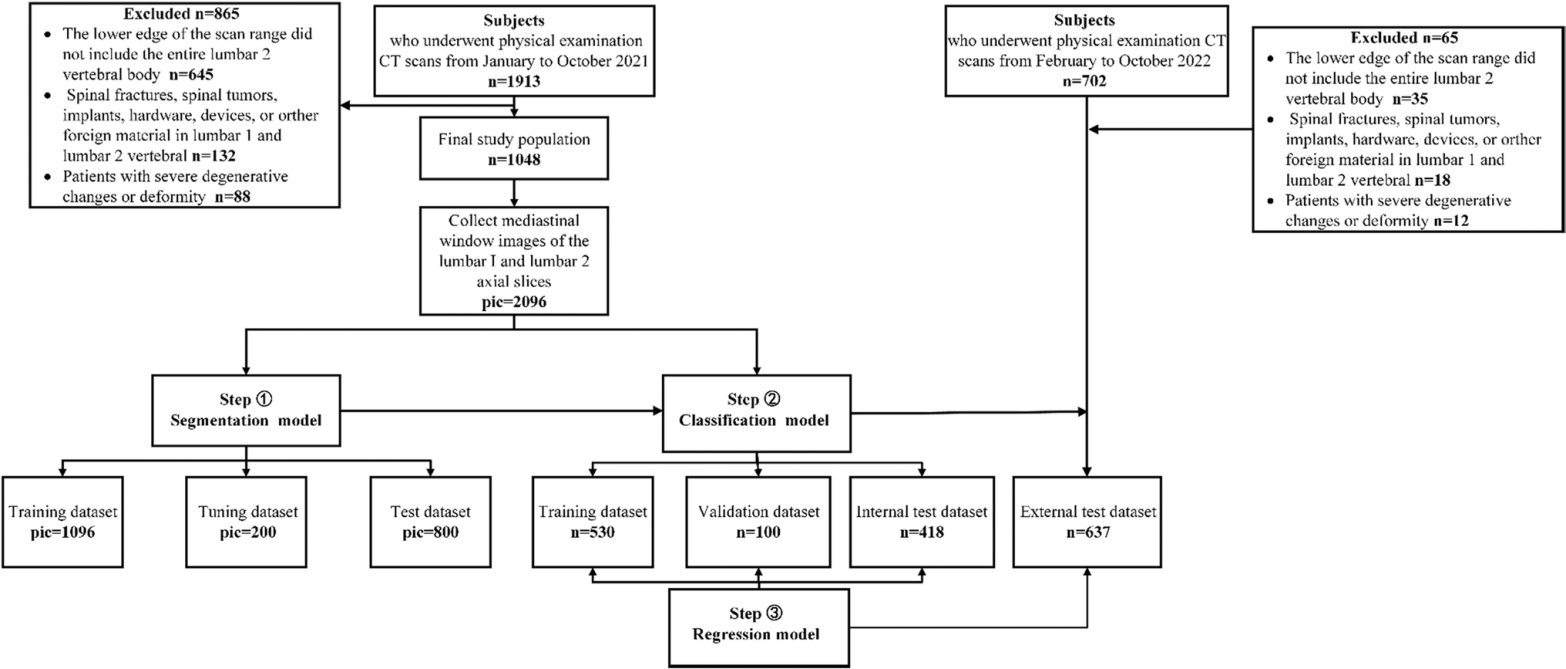
Flowchart for selecting the study population

A total of 1048 subjects were included in the internal dataset, and 637 subjects were selected for the external validation cohort. A pipeline depicting patient selection is displayed in Fig. 1.

### CT image acquisition

The CT scan parameters are shown in Appendix Table S1. BMD measurement and model construction were performed on the mediastinal window images of the center levels of the lumbar 1 and lumbar 2 vertebrae of each subject. We used 2D horizontal axis CT slices with a layer thickness of 2 mm.

### Bone mineral density measurement

QCT pro4 software (Mindways, CA, USA) was used to set similarly sized ROIs in the central cancellous bone region of lumbar 1 and lumbar 2, avoiding the cortical bone and the visible blood vessel area. The software automatically calculated the BMD values of the lumbar 1 and lumbar 2 vertebrae and used their mean values as the BMD values of the individual subjects (BMD_individual_). According to the criteria recommended by the guidelines [21], BMD_individual_ > 120 mg/cm^3^ was considered normal, 80 mg/cm^3^ ≤ BMD_individual_ ≤ 120 mg/cm^3^ was considered osteopenia, and BMD_individual_ < 80 mg/cm^3^ was considered osteoporosis.

## Model construction

We constructed three models in three steps: first, we constructed a segmentation model; then, we constructed a three‑classification model; and finally, we constructed a bone density prediction regression model.

### Image segmentation model

U-Net is a commonly used convolutional neural network architecture in medical image segmentation. The U-Net model was first proposed by Ronneberger et al. [22] in 2015. Based on U-Net, we design a new segmentation network, Bone-SegNet. First, the network depth is deepened, and the original coding layer and decoding layer of U-Net are both increased from four layers to seven layers. Deepening the network depth is helpful for extracting the lumbar spine image features.

For the five layers of the coding layer, a combination of a 1 × 1 convolution kernel and a 3 × 3 convolution kernel is included, the step size is 1, and the number of channels is 8, 16, 32, 64, and 128. In the sixth layer, the number of channels is 256, the size of the convolution kernel is 2 × 2 and 3 × 3, and the step size is 2 and 1. For the seventh layer, the number of channels is 512, the convolution kernel size is 2 × 2, and the step size is 2. In the decoding layer, the number of channels corresponds to the previous coding layer one to one, and the channel attention mechanism (CBAM) module is introduced into each layer of the decoding layer. The channel attention mechanism (CBAM) adds a global average pool (GAP) layer after adding the number of feature channels between high-level features and low-level features to reduce the dimension of the original feature map, and the dimension of the obtained feature map is *R*^*C*×1×1^. Different from SE-Net [23], the CBAM in this paper uses two 1 × 1 convolutional layers to replace the original two fully connected layers, which can focus on highlighting local edge information. The first convolutional layer uses ReLU as the activation function, and the second convolutional layer uses the sigmoid activation function to limit the feature weight range to [0,1]. The attention vector is obtained by multiplying the weight of each channel with the low-level feature map, and the attention feature map is obtained by adding the high-level feature map to improve the segmentation results. A skip connection layer is used to connect the corresponding encoding layer and decoding layer to help the decoding layer better obtain the details of the image. The size of the original CT image is *n* × 512 × 512 × 3, where *n* is the image batch size. The output image size of Seg-Net is the same as the input size.

The internal dataset (2096 images) was randomly divided into a training set (pic = 1096), tuning set (pic = 200), and testing dataset (pic = 800) at a 5:1:4 ratio. All 1274 images from the external validation cohort were used for external testing. An experienced radiologist with 3 years of experience in musculoskeletal imaging diagnosis manually drew the ROIs along the inner edge of the cortical bone at the central level of the lumbar 1 and lumbar 2 vertebrae with QuPath software (https://qupath.github.io/, version 0.4.4) to extract lumbar vertebral images. The dice similarity coefficient (DSC) was automatically calculated by the software to compare the consistency between the automatic segmentation of the DCNN and manual labelling. The stochastic gradient descent (SGD) optimizer plus Momentum mode was used for training; Momentum mode accelerates the updating of the SGD optimization algorithm at larger step lengths. The Momentum size is 0.9; the weight_decay size is 5e − 4; the number of training rounds is 500; the learning rates of the first 250 rounds and the second 250 rounds are 0.01 and 0.001, respectively; and the batch size is 32, producing the best model for the validation set.

### Clinical covariates

We collected some clinically significant risk factors for osteoporosis in the same period that CT scans were performed [24], including (1) general subject data: age, sex, height, weight, blood pressure (systolic blood pressure, diastolic blood pressure), and body mass index (BMI), calculated as weight (kilograms) divided by the square of the height (meters), and (2) laboratory biochemical indicators: triglycerides (TG), hemoglobin (Hb), fasting blood glucose (FBG), and total cholesterol (TC), as measured from the fasting venous blood.

### Radiomics feature selection

The CT images of the lumbar 1 and lumbar 2 vertebrae and the mask images obtained from the segmentation network were used as input to Python software, and the radiomics features were extracted by using the Pyradiomics software package (https://pyradiomics.readthedocs.io/en/latest/). A total of 868 features were extracted, including shape features of the original image and the wavelet transform, first-order features, and texture features (GLCM, grey-level size region matrix (GLSRM), grey-level running length matrix (GLRLM), adjacent grey-level difference matrix (AGLDM), and grey-level dependence matrix (GLDM)). To speed up the model training, improve the generalization of the model, and eliminate redundant features, we used the support vector machine (SVM) recursive feature elimination method to filter the extracted features. To account for the common influence of the two levels of vertebral bodies on the results, we extracted the radiomics features of the two levels of vertebral bodies and used the mean value as the basis for judging the results.

### Deep learning feature selection

In the task of image classification, when a Bone-ClassNet is used to extract high-level semantic features of the images, the resolution of the feature mapping can be reduced and more abstract features can be obtained only be increasing the depth of the network. Compared with other CNN networks, ResNet [25] has low complexity, and fewer parameters are required; additionally, the residual module weakens the vanishing gradient problem of the deep network. Since the convolution layer produces the output feature through a linear combination of the local convolution kernel and the original feature, Bone-ClassNet uses stacked convolution layers to increase the receptive field, but this method is often not efficient. Therefore, we introduced a self-attention mechanism [26] to capture global information to achieve a greater receptive field and contextual information. The specific network structure is shown in Appendix Table S2.

The CT image and mask image obtained by the segmentation network are processed together to obtain the corresponding segmentation image, which is used as the input of Bone-ClassNet. To remove useless features around the region of interest, we cropped the ROI and scale it to a size of 224 × 224, which is more appropriate for Bone-ClassNet. We chose ResNet-101 as the base architecture and maintained the number of original dense blocks at 3, 4, 23, and 3. Each dense block is stacked by 1 × 1 and 3 × 3 convolution kernels. The only difference is that we added a self-attention mechanism in front of each dense block. Finally, we flattened the feature map into one-dimensional vectors as the features for our deep learning. We used the Adam optimizer with a batch size set to 48, training rounds set to 300, and an initial learning rate of 0.01. For every 100 rounds of training, the learning rate was reduced by 0.1 from the original to produce the best model for the validation set.

### Multi-feature fusion classification model construction

To reflect the interaction of multiple features on the classification results, we used the features and clinical information after radiomics screening as input, constructed a new feature vector with the output of Bone-ClassNet, and classified it through a multi-layer perceptron composed of three fully connected layers and the Softmax classifier. Multiple fully connected layers can increase the nonlinearity of the model and improve the robustness of the classification model.

### Bone density prediction regression model

After the segmentation of lumbar images at the first lumbar vertebra levels (Bone-SegNet model) and the extraction of vertebral body regions of interest, we input the pre-processed samples into the Bone-ClassNet classification network. With this step, we get the forecast probabilities for three categories. Next, we combined these predicted probabilities with clinical indicators and CT values for regions of interest in the segmentation network to build a multi-layer perceptron fitting model of bone density with multi-feature inputs. The goal of this model is to accurately predict bone mineral density by taking into account multiple features such as clinical indicators, anatomical information of the region of interest of the segmentation network, and CT values. Dataset partitioning is the same as the classification model.

**Fig. 2:**
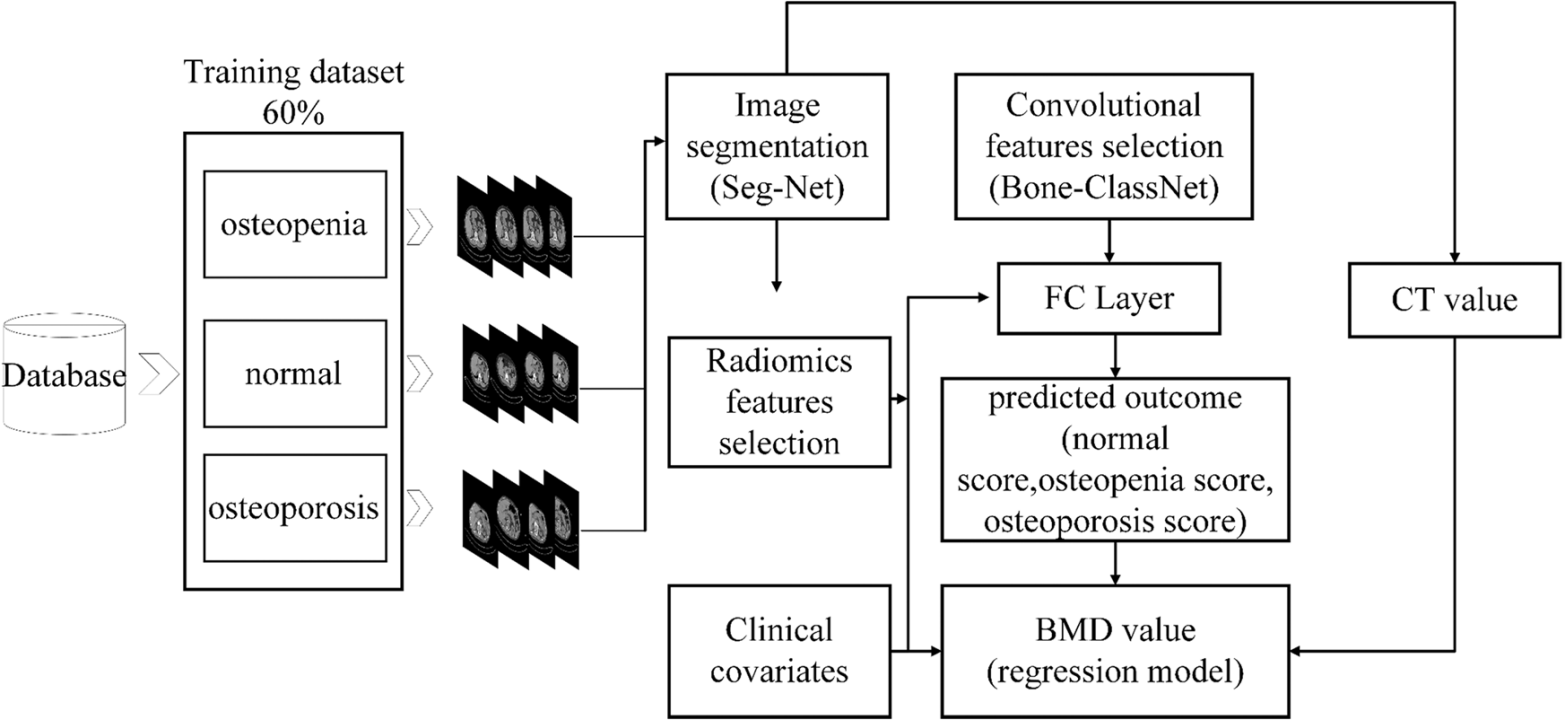
Bone density classification and bone density value prediction network training flowchart

First, we read the header information of DICOM image and extract the slope and intercept of all pixel data. Then, according to the coordinates of the region of interest in the segmentation image obtained in advance, the DICOM pixel data of the desired region is obtained by using these coordinates to calculate the CT value. Then, CT values, class probability scores, and some common clinical indicators were combined into a one-dimensional feature vector, and BMD was linearly fitted through the fully connected layer of the neural network. Each neuron can represent a linear function of the input feature vector, and by adjusting the weights and bias terms, the neural network can learn the nonlinear model that best fits the training data. The mean squared error (MSE) was used to evaluate the loss during the training process. Calculate the root mean squared error (RMSE) and mean absolute error (MAE) between the predicted bone density values and BMD_QCT_ for each model, select the optimal model, and evaluate it using the external testing dataset. Calculate the Pearson correlation coefficient, and plot the Bland–Altman plot and correlation coefficient plot. The workflow of bone density classification and bone density value prediction network is shown in Fig. 2.

## Statistical analyses

All statistical analyses were performed using Python 3.6.12, SPSS 27, and R64 software. The receiver operating characteristic (ROC) curve was used to analyze the evaluation efficacy of each model on bone mass. The AUC and 95% confidence interval, sensitivity (Se), specificity (Sp), positive predictive value (PPV), negative predictive value (NPV), and accuracy (Ac) were calculated from ROC analysis. The Delong test was used to compare the difference in bone mass evaluation efficiency for testing datasets. Decision curve analysis (DCA) was used to evaluate the value of the model in clinical decision-making. When analyzing the influencing factors of osteoporosis, the classification of BMD was used as the dependent variable (control = normal bone mass), and univariate and multivariate analyses were performed by disordered multinomial logistic regression analysis. Spearman correlation was used to perform the correlation of variables with BMD. To assess odds ratios (ORs) and 95% confidence intervals for related variables according to the occurrence of osteoporosis, we used logistic regression analysis. Numerical data are expressed as the mean ± standard deviation $\left(\overline{x }\pm s\right)$, and comparisons between groups were performed with variance analysis or the *t* test. Classification data are expressed as the frequency and percentage (*n*, %), and the chi-square test was used for comparisons between groups. *P* < 0.05 was considered statistically significant.

## Results

### Baseline and clinical characteristics of collected data

Among 1685 participants (mean age 54.64 ± 15.31 years, ranged 20–92 years), 874 participants were male, and 811 participants were female. There were 844 patients with normal bone density status (mean BMD was 158.93 ± 26.02 mg/cm^3^, ranging from 120.30 to 275.10 mg/cm^3^), 503 with osteopenia (mean bone mineral density was 95.90 ± 10.93 mg/cm^3^, ranging from 80.00 to 120.00 mg/cm^3^), and 338 with osteoporosis (mean bone mineral density was 55.42 ± 17.35 mg/cm^3^, ranging from 6.70 to 79.90 mg/cm^3^).

Table 1 lists the subject distribution and baseline characteristics of the training, validation, and internal and external testing datasets. The results show that there was no significant difference in any baseline variable except systolic blood pressure in the internal datasets (training, validation, and internal testing datasets), which was homogeneous and comparable, facilitating our subsequent analysis.

**Table 1:** Distribution of subjects and baseline characteristics in each dataset

Characteristics		Internal testing dataset						External testing dataset		
		Overall *n* = 1048	Training dataset *n* = 530	Validation dataset *n* = 100	Internal testing dataset *n* = 418	*F*	*P*	External testing dataset *n* = 637	*F*	*P*
Gender *n*, %	Male	605, 57.73%	305, 57.55%	62, 62.00%	238, 56.94%	0.862*	0.650	270, 42.39%	26.60*	< 0.001
	Female	443, 42.27%	225, 42.45%	38, 38.00%	180, 43.06%			367, 57.61%		
Age (yr.)^#^		51.19 ± 14.35	51.04 ± 14.4	51.11 ± 12.73	51.70 ± 14.97	0.255	0.775	67.59 ± 9.11	9.904	< 0.001
Height (cm)^#^		165.00 ± 8.28	164.76 ± 8.23	165.31 ± 8.56	165.22 ± 8.30	0.425	0.854	162.74 ± 7.89	3.865	0.963
Weight (kg)^#^		67.31 ± 12.06	67.21 ± 12.00	67.45 ± 11.76	67.41 ± 12.24	0.035	0.966	66.32 ± 12.04	5.955	0.701
BMI (kg/m^2^)^#^		24.62 ± 3.38	24.66 ± 3.47	24.55 ± 2.91	24.59 ± 3.37	0.061	0.940	24.48 ± 4.76	3.140	0.022
BMD (mg/cm^3^)^#^		130.28 ± 42.76	129.92 ± 42.78	131.30 ± 39.29	130.90 ± 42.96	0.084	0.919	131.36 ± 43.49	0.148	0.050
Blood pressure (mmHg)^#^	SBP	126.07 ± 32.59	125.1 8 ± 19.23	136.20 ± 85.80	124.68 ± 18.98	5.437	0.004	126.22 ± 13.70	5.276	0.050
	DBP	76.94 ± 10.61	76.71 ± 10.92	78.89 ± 10.79	76.94 ± 10.14	1.883	0.153	75.54 ± 7.56	3.865	0.692
Triglycerides (mmol/L)^#^		2.03 ± 8.13	1.77 ± 1.53	1.95 ± 1.49	2.38 ± 12.69	0.648	0.523	2.04 ± 4.933	0.157	0.015
Hemoglobin (g/L)^#^		145.00 ± 16.56	144.98 ± 16.32	146.95 ± 14.85	144.57 ± 17.24	0.827	0.437	136.61 ± 17.08	49.111	< 0.001
FBG (mmol/L)^#^		5.69 ± 1.40	5.68 ± 1.25	5.71 ± 1.40	5.68 ± 1.56	0.022	0.978	6.27 ± 2.20	16.020	< 0.001
TC (mmol/L)^#^		4.71 ± 0.88	4.69 ± 0.89	4.69 ± 0.99	4.74 ± 0.85	0.325	0.723	4. 47 ± 1.02	0.002	0.077
BMD categories, *n*, %	Normal	621, 59.64%	310, 58.49%	60, 60.00%	251, 60.05%	0.994*	0.911	223, 35.00%	81.94*	< 0.001
	Osteopenia	296, 27.96%	150, 28.30%	30, 30.00%	116, 27.75%			207, 32.50%		
	OP	131, 12.40%	70, 13.21%	10, 10.00%	51, 12.20%			207, 32.50%		

χ^2^ test and Fisher’s exact test for categorized variables

# Data is represented by mean ± SD

* For chi-square test; *BMI*, Body mass index; *BMD, *Bone mineral density; *SBP,* Systolic blood pressure; *DBP,* Diastolic blood pressure; *FBG, *Fasting blood-glucose; *TC, *Total cholesterol; *OP*, osteoporosis

Correlation analysis showed that osteoporosis was positively correlated with sex (*P* < 0.01) and negatively correlated with age (*P* = 0.040), height (*P* < 0.01), weight (*P* < 0.01), BMI (*P* = 0.039), diastolic blood pressure (*P* = 0.040) and hemoglobin (*P* < 0.01). A plot of the correlation coefficients is shown in Appendix Fig. S1.

Univariate logistic regression analysis showed that osteoporosis was related to total cholesterol and especially age. In order to reasonably estimate and interpret the regression model, collinearity diagnosis was carried out for factors (age and total cholesterol) of significance in univariate analysis. The results show that the variance inflation factor (VIF) value of age and total cholesterol is 1.004, and the tolerance is 0.996. It can be considered that age and total cholesterol do not have collinearity. Multivariate multiple logistic regression analysis was then performed on these two factors and showed that people of older age and with higher cholesterol levels were more likely to have osteoporosis.

### Segmentation results

When the number of images in the training set was greater than 300, the value of DSC (0.95 ± 0.004) tended to be stable. When the number of images in the training set was 300, the DSC value was 0.951 ± 0.03 in the testing dataset and 0.947 ± 0.060 in the external validation set. This is sufficient to ensure the performance of vertebral image segmentation. The segmentation effect of some CT images is shown in Appendix Fig. S2, where it can be seen that Bone-SegNet had good segmentation accuracy and obvious advantages in maintaining the shape of different lumbar vertebrae and could accurately identify the position of the lumbar vertebrae, confirming that Bone-SegNet was effective for our segmentation task.

### Classification results

The main dataset was constructed from the CT images of 630 subjects from January 2021 to February 2021 (randomly divided into a training dataset and validation dataset at a ratio of 5:1). The remaining 418 patients comprised the testing dataset. A total of 637 patients were selected for the external validation cohort.

#### Radiomics feature selection

**Fig. 3:**
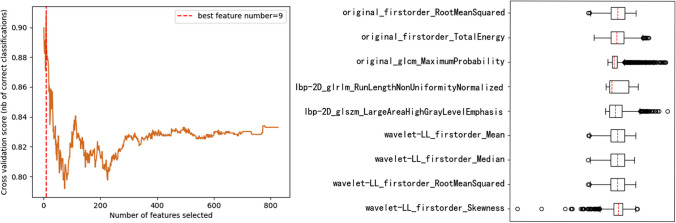
Support vector machine recursive feature screening diagram and boxplot of nine omics features

To screen for the most useful radiomics features, we adjusted the best feature vector through cross-validation automatically and screened the cross-validation scores for different feature numbers, as shown in Fig. 3; the highest score was obtained for nine features, whose data were visualized with boxplots. It can be seen from the diagram that except for original_glcm_Maximum Probability, lbp-2D_glszm_LargeAreaHighGrayLevelEmphasis, and wavelet-LL_firstorder_Skewness—which had a certain number of outliers—none of the other features had substantial outliers; regardless, given the large size of our sample dataset, these outliers had little impact on the calculation and analysis of the data.

#### Prediction performance evaluation

A total of eight models were established, namely, (1) a radiomics model based on random forest; (2) a radiomics model based on naive Bayes; (3) a DCNN model based on Bone-ClassNet; (4) a fusion model based on radiomics features and deep learning; (5) a multi-feature fusion model based on clinical information and deep learning; (6) a multi-feature fusion model based on clinical information, radiomics features, and deep learning; (7) a model derived from model 3 constructed by using the lumbar 1 vertebral images alone; and (8) a model based on model 6 constructed by the lumbar 1 vertebral images alone. The model specific information is shown in Appendix Table S3. The evaluation efficacy of the different models on bone mass in each dataset is shown in Appendix Table S4.

In all models, the multi-feature fusion model (model 8) demonstrated the best performance, with AUC score values of 0.992, 0.973, and 0.989. The sensitivity of this model exceeds 0.90, which is suitable for screening and guiding the next clinical diagnosis and treatment. The specificity is also high, and it is also suitable for diagnosis.

Compared to model 8, although the AUC values of model 2 and model 4 were higher than those of model 8 (0.995 vs. 0.992, 0.993 vs. 0.992, respectively) in diagnosing normal bone mass, there was no significant difference among these models (*P* = 0.089 and *P* = 0.989). Model 8 in diagnosing osteopenia was the best with the AUC value = 0.973. Although the AUC values of models 1 and 2 were higher than those of model 8 (both 0.993 vs. 0.989) in diagnosing osteoporosis, the differences were not statistically significant (*P* = 0.356 and *P* = 0.187) (Appendix Table S4).

Based on the above reasons, we chose model 8 as the best model to calculate the AUC value of the external test set and establish the subsequent BMD regression model. The AUC of the multi-feature fusion model established with the lumbar 1 vertebral body in the diagnosis of normal, osteopenia, and osteoporosis in the external dataset was 0986, 0.930, and 0.975, respectively (Appendix Table S4).

#### Clinical use

The Delong test showed that the differences in the AUCs between the multi-feature fusion model based on the images of lumbar vertebrae 1 (model 8) and the Bone-ClassNet model based on lumbar vertebrae 1 and 2 (model 3) were not significant regardless of bone density status (Appendix Table S5).

Decision curve analysis was used to compare model 8 and model 3 in the testing dataset.

**Fig. 4:**
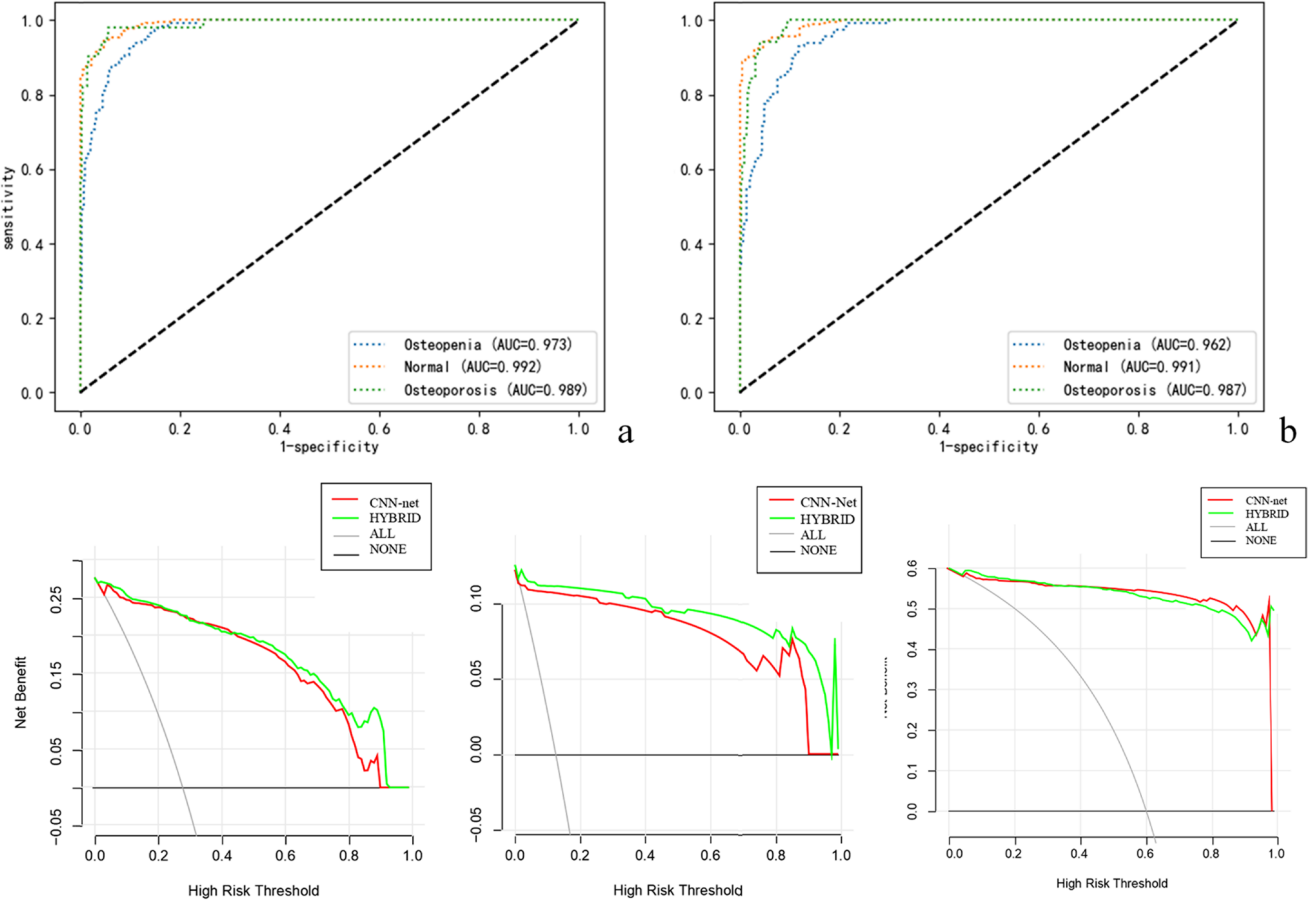
Comparison of AUC curve and DCA curve of Model 3 and model 8. **a**, **b** The ROC curves of the multi-feature fusion model 8 and the Bone-ClassNet model 3 to diagnose normal, osteopenia, and osteoporosis. **c**–**e** The DCA curves revealed that model 8 (green line) was more advantageous than model 3 (red line). The *x*-axis corresponds to the threshold probability while the *y*-axis corresponds to the net benefit. The gray line represents the assumption that all lesions are due to osteoporosis. The black line indicates that no lesion is due to osteoporosis

For the normal bone mass group, if the clinical decision-making threshold probability is greater than 40%, the multi-feature fusion model has more clinical benefits than the independent Bone-ClassNet model. Within this range, the net benefits of the two models are comparable, but the multi-feature fusion model performs better. For osteopenia, the multi-feature fusion model outperforms the Bone-ClassNet model across all threshold probability intervals. For osteoporosis, the two curves are similar, and the net benefit rates are much higher than 0, indicating that both prediction models have certain clinical utility. The decision curve analysis plot of the models is shown in Fig. 4.

### Performance evaluation of bone density prediction model

**Fig. 5:**
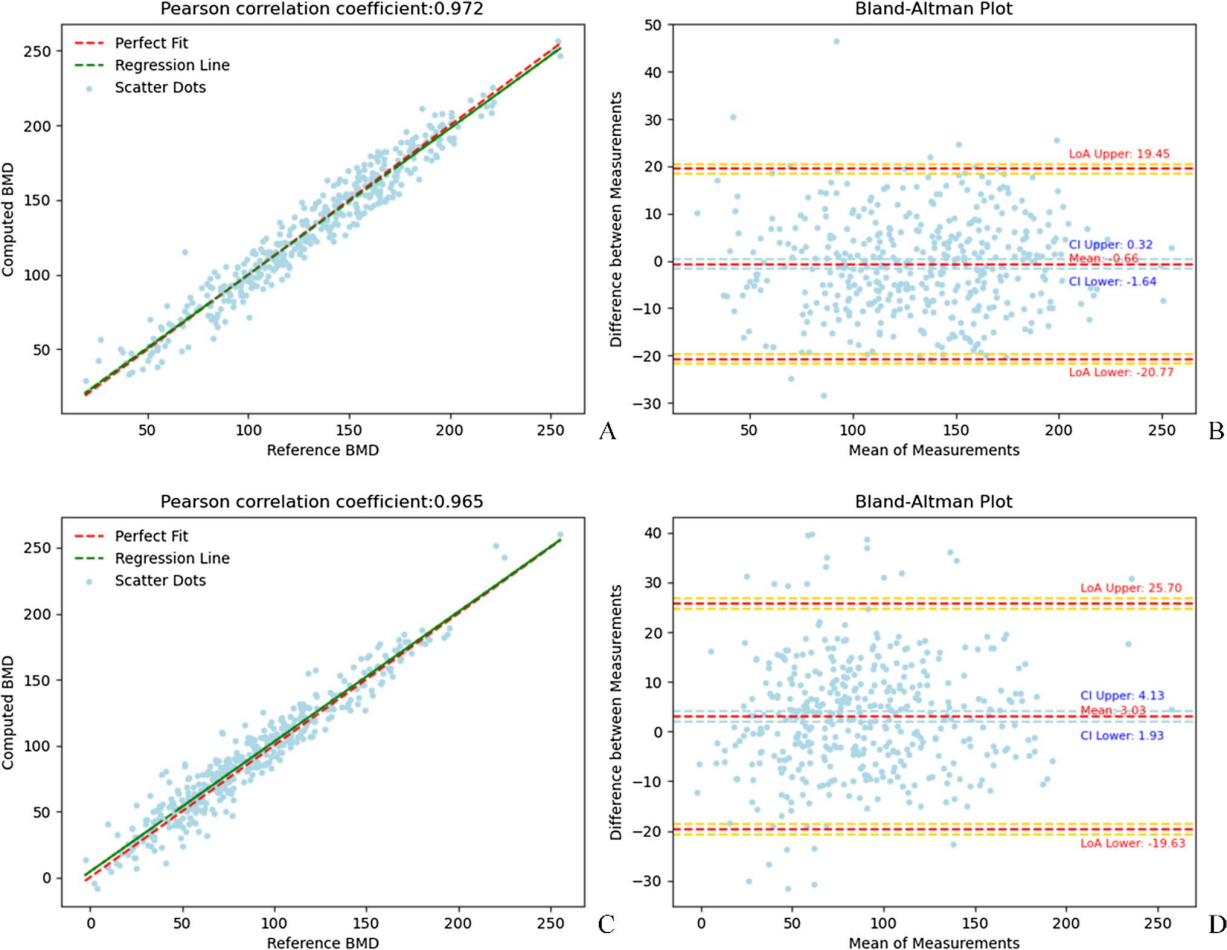
Correlation plot and Bland–Altman plot of the test set and external testing dataset. A scatter plot of BMD_QCT_ and predicted BMD in the test set (**A**, **B**) and external testing dataset (**C**, **D**). The horizontal axis shows BMDQCT. The vertical axis shows predicted BMD. Correlation plot with confidence interval set at 95% (shadowed area). Red line represents reference line; blue line is regression line. Blue crosses are distribution dots. The Pearson correlation coefficient for the test set is 0.972; for the external testing dataset, it is 0.965. The *R*-squared value for the test set is 0.942; for external testing dataset, it is 0.923. In the Bland–Altman plot, the horizontal axis shows the average of the results for each sample measured by the two methods, and the vertical axis shows the difference between the two methods. The limits of agreement (LoA) are shown as dashed orange lines with 95% confidence intervals (light orange areas), while the bias is shown as a dashed blue line with a 95% confidence interval (light blue area). The degree of agreement between the two measurements is reflected by the tightness of the distribution of points around the central mean line. Outliers are observations that lie above and below the light red bands, respectively. In the Bland–Altman plot of the test set, the mean difference is 0.66, the higher LoA is 19.45, the lower LoA is - 20.77, and 1.67% (7/418) of the points are outside the 95% LoA. For the external testing dataset, the mean difference is 3.03, the higher LoA is 25.70, the lower LoA is - 19.63, and 5.24% (22/386) scatters are outside the 95% LoA

The network was trained for 500 iterations with mean square error as the loss function. After over 500 iterations, the loss became stable. The MAEs of the bone density prediction regression model in the test set and external testing dataset are 8.20 mg/cm^3^ and 9.23 mg/cm^3^, respectively. The RMSEs for the same sets are 10.25 mg/cm^3^ and 11.91 mg/cm^3^, respectively. The *R*-squared values are 0.942 and 0.923, respectively. The Pearson correlation coefficients (*ρ*) are 0.972 and 0.965. Figure 5 shows the Bland–Altman plot and correlation coefficient plot of the bone density values predicted by the model relative to BMD_QCT_.

We selected the mediastinal window images of the center levels of the vertebral images on chest CT, and then, the model was run completely automatically for segmentation, classification, and bone mineral density calculation.

## Discussion

In this study, we firstly used opportunistic CT scan data from the chest CT images and applied automatic segmentation to obtain the ROI for trabecular bone. Then, we developed a multi-feature fusion three-classification model using features from three different sources, namely, (1) clinical information, (2) radiomics features, and (3) a deep learning model for bone density and a regression model for predicting bone density values. The experimental results demonstrated that the three-classification model accurately classified the trabecular bone density of the spine, and the bone density value prediction regression model accurately predicted the trabecular bone density values of the spine.

This study demonstrates that the multi-feature fusion DCNN classification model based on CT images of lumbar vertebra 1 was the best model. The AUCs of normal bone mass, osteopenia, and osteoporosis were 0.992, 0.973, and 0.989, respectively. BMD value prediction can more accurately reflect bone density. The MAEs of the bone density prediction regression model in the test set and external testing dataset are 8.20 and 9.23, respectively. The RMSEs for the same sets are 10.25 and 11.91, respectively. The *R*-squared values are 0.942 and 0.923, respectively. The Pearson correlation coefficients (*ρ*) are 0.972 and 0.965.

Few articles have investigated the diagnosis of osteoporosis via a multi-feature fusion model based on deep learning, radiomics, and clinical information. The results of this study show that sex, age, height, weight, BMI, diastolic blood pressure, and hemoglobin have significant effects on the prevalence of osteoporosis. The results of multiple linear regression showed a significant negative correlation between bone density status and age, consistent with previous literature [27, 28]. We found that the addition of patient clinical covariates provided more important information for classification and yielded improved performance metrics over the image-only model. Specifically, compared with the model with image-only information, the model that incorporated clinical information had significantly improved performance in diagnosing osteopenia (0.970 vs. 0.951, *P* = 0.03) and similar performance in diagnosing normal (0.990 vs. 0.993, *P* = 0.06) and osteoporosis (0.989 vs. 0.988, *P* = 0.30). Furthermore, the sensitivity, specificity, and other performance metrics were improved. Additionally, we performed external validation using data acquired from a different vendor and achieved good diagnostic performance, demonstrating that our model has good versatility and generalizability. Fang et al. used a regression model based on DenseNet to predict BMD values for some cases in three testing cohorts, with *R*^2^ ranging from 0.780 to 0.948, similar to this study [16]. Pan et al. also predicted BMD values using a deep learning model, with *R*^2^ ranging from 0.964 to 0.968. Bland–Altman analysis also showed good consistency between the predicted BMD values and QCT reference values, consistent with the results of this study [29].

BMD measurement is an important basis for the diagnosis, risk prediction, and efficacy evaluation of osteoporosis [30]. QCT is a three-dimensional imaging technology that can quantitatively evaluate the vertebral cancellous bone mineral density, is more sensitive, and produces more stable measurement results than DXA [7]. In addition to bone mineral density, bone microstructure geometric features and density structure changes can also reflect the degree of osteoporosis to a certain extent [31], manifesting as changes in local image features in CT images. Trabecular thinning and trabecular loss can be observed in cancellous bone. In men, it is mainly related to reduced bone formation and low bone turnover. Changes in bone matrix and mineral composition can also lead to increased bone fragility; this is why the nine originally extracted radiomics features mainly consisted of wavelet features [32] and texture features [13], including GLCM and GLSZM features. These features can comprehensively reflect the spatial heterogeneity of the vertebral body.

Single-image features only represent the internal features of the structure, while the multi-dimensional source data can comprehensively evaluate the state of the body and provide more abundant feature information. Previous studies have also shown that models constructed by integrating clinical, pathological, and image features are significantly superior to single-parameter models. Sukegawa et al. [33] used an integrated deep learning model based on dental panoramic X-ray film and clinical information to diagnose osteoporosis, with significantly greater AUC values than an image-only model (0.845 vs. 0.832; 0.921 vs. 0.900). Luo et al. [34] first established a multi-channel convolutional neural network (MCNN) diagnostic model for osteoporosis by combining age, height, weight, and quantitative ultrasound, and the sensitivity, specificity, and accuracy in diagnosing osteoporosis were 80.86%, 84.23%, and 83.05%, respectively. Xie et al. [35] used QCT to screen 590 patients with low bone mass or osteoporosis, collected clinical information, and used patient CT images of the third lumbar spine to construct a combined clinical-radiomics model. The model combined radiomics and clinical features (age, alkaline phosphatase, and homocysteine), and the AUC was 0.96.

In this study, we explored the possibility of constructing a model with lumbar 1 vertebra alone to diagnose osteoporosis. In general, the lower boundary of both lungs is located at the level of the thoracic 12 vertebrae [36], so using lumbar 1 and lumbar 2 images based on the chest CT to construct a diagnostic model to assess BMD requires the technologists to expand the scanning length so that the lower boundary of the scanning range includes the lumbar 2 vertebra. We constructed a diagnostic model based only on lumbar 1 vertebral body images. The benefits of doing this are obvious: firstly, moving up the lower limit of the CT scanning range from the level of the lumbar 2 vertebra to the level of the lumbar 1 vertebra shortens the scanning length of the *z*-axis and reduces the patient’s radiation exposure. Although the reduction of radiation dose may be limited for a single individual, considering the huge number of annual health check population, this change is quite significant in terms of reducing the overall radiation dose of the population. Secondly, since there is no need to intentionally expand the scanning range and require additional requirements or training for technologist, they can perform chest CT scanning according to their usual habits, thereby avoiding additional training costs. Finally, using a single-image-based DCNN model for opportunistic osteoporosis screening has the potential to reduce the data collection costs of the model and alleviate the storage pressure on the post-processing workstation. In fact, before we started our research work, there was someone had attempted to use imaging information from a single vertebral body for the diagnosis of osteoporosis. Pickhardt et al. [37] used 1867 adults undergoing CT and DXA over 10 years with 90% sensitive and more than 90% specific for distinguishing osteoporosis from osteopenia and normal BMD at L1 CT-attenuation threshold of 110 HU. This successful result at least indicates that we are not alone on the path of using single vertebral body–related information for osteoporosis screening. However, focusing on accuracy alone cannot provide a comprehensive assessment of the model. A predictive model with a higher AUC may have high specificity but low sensitivity, so it cannot be applied in practice. Decision curve analysis [38] is a simple and easy-to-understand mathematical technique for evaluating the suitability and effectiveness of the prediction model for use in the clinic; by analyzing all possible behaviors and results, the optimal behavior of the model is selected. DCA showed that compared with the simple Bone-ClassNet model, the multi-featured fusion model based on lumbar 1 vertebral image had better predictive performance and greater clinical benefits.

## Limitations

Although the proposed method achieved convincing results, some limitations should be mentioned.

Firstly, the study uses Mindways-based QCT as gold standard. Mindways has its own limitations and error sources which limits its use a gold standard. More generally speaking, in most countries, DXA but not QCT is recognized to define osteoporosis and osteopenia. The ability of chest CT–based AI models to estimate DXA-based osteoporosis and osteopenia or areal BMD was not studied in this contribution. Secondly, evaluating only a single vertebra may be less robust compared to an evaluation of several vertebrae, particularly if L1 is beginning to show signs of fracture. Thirdly, because the cohort was recruited from the general annual health check population, there was an imbalance in the number of subjects across the osteoporosis, osteopenia, and normal BMD groups. This imbalance may have resulted in a lower PPV in the validation set. Fourthly, this study only constructed a DCNN model for two-dimensional axial CT slices with a layer thickness of 2 mm and did not include all the three-dimensional image features of the target vertebral body. However, the two-dimensional model has a simple structure, low data demand, low computational complexity, and short training time, which is conducive to rapid deployment in clinical practice. Finally, we collected some recognized factors, such as lipid biomarkers, that were not significantly correlated with osteoporosis except the total cholesterol level, which is positively correlated with the incidence of osteoporosis, consistent with previous studies [39, 40]. The inconsistent results of sex, weight, BMI, and other factors with those of previous studies [41] may be due to the lack of stratified analysis of these indicators in this experiment, and the effectiveness of these factors needs to be further verified. Additionally, few clinical features were included in the present study, only age, sex, BMI, etc. In future investigations, we will seek to address these limitations.

## Conclusion

In conclusion**,** the multi-feature fusion DCNN model based on only the lumbar 1 vertebrae and clinical variables can perform bone density three-classification diagnosis and estimate volumetric bone mineral density. If confirmed in independent populations, this automated opportunistic chest CT evaluation can help clinical screening of large-sample populations to identify subjects at high risk of osteoporotic fracture.

## Supplementary Information

Below is the link to the electronic supplementary material.

Supplementary file1 (DOCX 684 KB)

Supplementary file2 (PDF 793 KB)

## Data Availability

The datasets are not publicly available because of restrictions in the data-sharing agreements with the data sources. The data that support the findings of this study are available from the corresponding author upon reasonable request.

## Abbreviations

AUC: Area under the curve
BMD: Bone mineral density
CI: Confidence interval
CNN: Convolutional neural network
DCA: Decision curve analysis
DCNN: Deep convolutional neural network
DXA: Dual-energy X-ray absorptiometry
FOV: Field of view
L1: Lumbar 1 vertebra
L2: Lumbar 2 vertebra
LoA: Limits of agreement
MAE: Mean absolute error
MSE: Mean square error
QCT: Quantitative computed tomography
ROC: Receiver operating characteristic
ROI: Region of interest
RMSE: Root mean square error
SCOOP: Screening for prevention of fractures in older women
SGD: Stochastic gradient descent
WHO: World Health Organization
