# Supplementary material for: Feasibility study of opportunistic osteoporosis screening on chest CT using a multi-feature fusion DCNN model
Source: Arch Osteoporos. 2024 Oct 17;19(1):98. doi: 10.1007/s11657-024-01455-7 (PMC11485148; doi:10.1007/s11657-024-01455-7)
Supplement: Supplementary file 1 — Supplementary file1 (DOCX 684 KB) [file 11657_2024_1455_MOESM1_ESM.docx]

**Supplementary document/ Appendix**

**Appendix Table S1**.  **Details of CT protocols**

| **CT information** | | **Parameters** | |
| --- | --- | --- | --- |
|  |  | **Internal scanner** | **External scanner** |
| **CT system** |  | Ingenuity Core 128 CT | SOMATOM Force CT |
| **CT scan** | Tube voltage | 120kVp | 120kVp |
|  | Time–current product | iPatient  range: 100~300 mAs | CARE Dose4D |
|  | Detector collimation | 64×0.625mm | 192×0.6mm |
| **CT image** | Image matrix | 512×512 | 512×512 |
|  | Field of view | 500×500mm | 500×500mm |
|  | Reconstruction section thickness | 2.0mm | 2.0mm |

**Appendix Table S2**. **Bone-ClassNet structure diagram**

| **Layer Name** | **Output Size** | **Architecture** |
| --- | --- | --- |
| Conv 1 | 112×112 | 7×7,64, stride 2 |
| Conv 2_x | 56×56 | 3×3, maxpool, stride 2 |
|  |  | $\left[ \begin{aligned} \text{1×1,64} \\ \text{3×3,64} \\ \text{1×1,}\text{256} \end{aligned} \right]$×1＋$\left[ \begin{aligned} \text{1×1,64} \\ \text{3×3,64} \\ \text{1×1,}\text{256} \end{aligned} \right]\text{×2}$ |
|  |  | 1×1,64, stride 1 |
| Conv 3_x | 28×28 | $\left[ \begin{aligned} \text{1×1,128} \\ \text{3×3,128} \\ \text{1×1,512} \end{aligned} \right]$×1＋$\left[ \begin{aligned} \text{1×1,128} \\ \text{3×3,128} \\ \text{1×1,}\text{512} \end{aligned} \right]\text{×3}$ |
|  |  | 1×1,512, stride 1 |
| Conv 4_x | 14×14 | $\left[ \begin{aligned} \text{1×1,256} \\ \text{3×3,256} \\ \text{1×1,1024} \end{aligned} \right]$×1＋$\left[ \begin{aligned} \text{1×1,256} \\ \text{3×3,256} \\ \text{1×1,1024} \end{aligned} \right]\text{×22}$ |
|  |  | 1×1,1024, stride 1 |
| Conv 5_x | 7×7 | $\left[ \begin{aligned} \text{1×1,512} \\ \text{3×3,512} \\ \text{1×1,2048} \end{aligned} \right]$×1＋$\left[ \begin{aligned} \text{1×1,512} \\ \text{3×3,512} \\ \text{1×1,2048} \end{aligned} \right]\text{×2}$ |
|  |  | 1×1, 2048, stride 1 |
|  | 1×1 | Average pool,2048-d  fc, softmax |

**
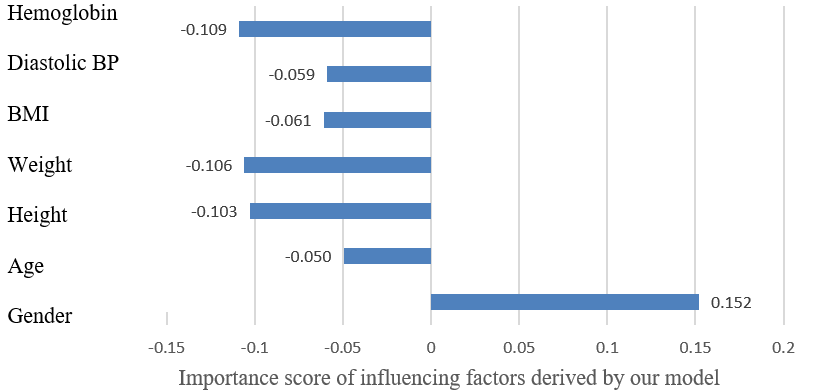
**

**Appendix Fig. S1.** 7 Influencing factors of Osteoporosis.


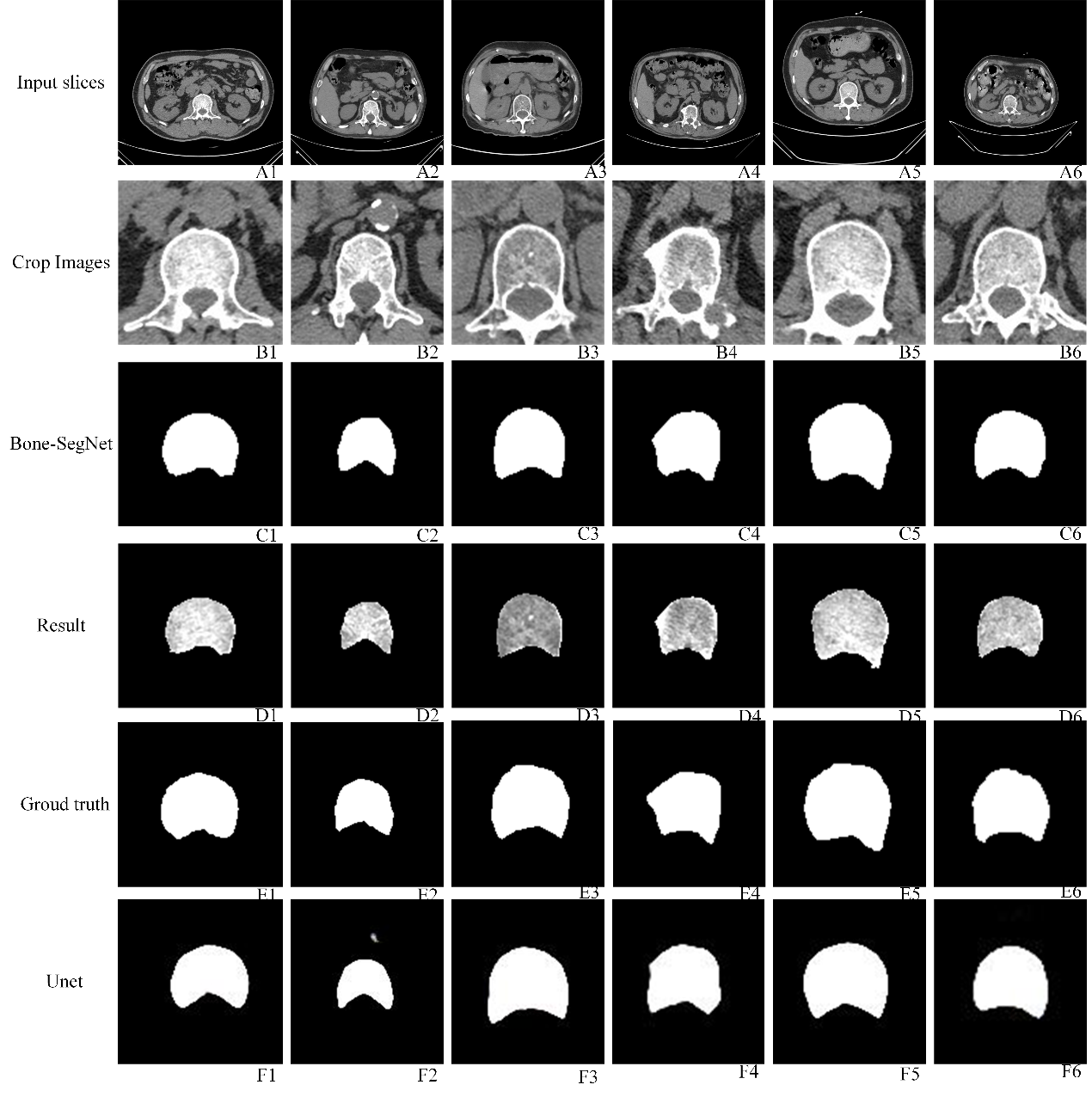


**Appendix Fig.S2.** Partial segmentation results. Fig. A1-F1: The female was 53 years old, who was diagnosed as normal bone mass with BMD _individual_=140.7mg/cm^3^. Fig. A2-F2: The male was 81 years old, who was diagnosed as normal bone mass with BMD _individual_=133.8mg/cm^3^. Fig. A3-F3: The female was 64 years old, who was diagnosed as osteoporosis with BMD _individual_=54.3mg/cm^3^. Fig. A4-F4: The male was 67 years old, who was diagnosed as osteoporosis with BMD _individual_= 77.5 mg/cm^3^. Fig. A5-F5: The male was 50 years old, who was diagnosed as osteopenia with BMD _individual_=116.4 mg/cm^3^. Fig. A6-F6: The male was 73 years old, who was diagnosed as osteopenia with BMD _individual_= 102 mg/cm^3^. Fig. A1-A6: The axial CT images of the central slice of lumbar 1 vertebral body of 6 subjects successively. Fig.B1-B6: Local magnification of the vertebral body in order to see the effect of the network; Fig. C1-C6: Bone-SegNet segmented images. Fig. D1-D6: Extracted ROI. Fig. E1-E6: Groud truth images. Fig. F1-F6: U-net segmented image.

**Appendix Table S3. Introduction of eight classification models**

| **Model name** | **Model type** | **Model description** | **Data source** |
| --- | --- | --- | --- |
| Model 1 | radiomics model, based on random forest classifier | ML model (nonlinear tree-based model) integrated learning method | L1 + L2 |
| Model 2 | radiomics model, based on naive Bayes | ML model based on Bayes theorem and independent assumption of feature conditions | L1 + L2 |
| Model 3 | DCNN model, based on Bone-ClassNet | convolutional neural network (CNN) | L1 + L2 |
| Model 4 | fusion model, radiomics features + DL | filtered omics features were fused with the DL features extracted by CNN-Net,  and the full connection layer was used for classification | L1 + L2 |
| Model 5 | multi-feature fusion model  clinical information + DL | DL features extracted from CNN-Net and clinical information were fused,  and the full connectivity layer was used for classification | L1 + L2 |
| Model 6 | multi-feature fusion model  clinical information + radiomics features + DL | filtered omics features, the DL features extracted by CNN-Net and clinical information were fused, and the full connectivity layer was used for classification | L1 + L2 |
| Model 7 | DCNN model, based on Bone-ClassNet | extracts DL features through CNN, uses the full connection layer for classification | L1* |
| Model 8 | multi-feature fusion model  clinical information + radiomics features + DL | integrates the filtered omics features, the DL features extracted by CNN-Net and clinical information, and uses the full connectivity layer for classification | L1* |

**Appendix Table S4.** **Effectiveness evaluation of different models on the testing datasets**

| categories | Se | Sp | PPV | NPV | Ac | AUC |
| --- | --- | --- | --- | --- | --- | --- |
| Model 1 | | | | | | |
| normal | 0.932 | 0.982 | 0.987 | 0.906 | 0.952 | 0.991 |
| osteopenia | 0.862 | 0.934 | 0.833 | 0.946 | 0.914 | 0.966 |
| osteoporosis | 0.941 | 0.964 | 0.787 | 0.991 | 0.962 | 0.993 |
| Model 2 | | | | | | |
| normal | 0.876 | 0.994 | 0.995 | 0.842 | 0.923 | 0.995 |
| osteopenia | 0.853 | 0.891 | 0.750 | 0.941 | 0.880 | 0.951 |
| osteoporosis | 0.961 | 0.956 | 0.753 | 0.994 | 0.956 | 0.993 |
| Model 3 | | | | | | |
| normal | 0.900 | 0.982 | 0.986 | 0.868 | 0.933 | 0.991 |
| osteopenia | 0.905 | 0.891 | 0.760 | 0.961 | 0.894 | 0.968 |
| osteoporosis | 0.843 | 0.978 | 0.843 | 0.978 | 0.962 | 0.987 |
| Model 4 | | | | | | |
| normal | 0.920 | 0.964 | 0.975 | 0.889 | 0.938 | 0.993 |
| osteopenia | 0.905 | 0.927 | 0.826 | 0.962 | 0.899 | 0.951 |
| osteoporosis | 0.961 | 0.986 | 0.907 | 0.995 | 0.962 | 0.988 |
|  | Model 5 | | | | | |
| normal | 0.916 | 0.988 | 0.991 | 0.887 | 0.916 | 0.986 |
| osteopenia | 0.922 | 0.921 | 0.817 | 0.967 | 0.883 | 0.948 |
| osteoporosis | 0.941 | 0.980 | 0.873 | 0.992 | 0.966 | 0.986 |
| Model 6 | | | | | | |
| normal | 0.924 | 0.976 | 0.983 | 0.896 | 0.938 | 0.990 |
| osteopenia | 0.931 | 0.934 | 0.844 | 0.972 | 0.914 | 0.970 |
| osteoporosis | 0.980 | 0.989 | 0.926 | 0.997 | 0.976 | 0.989 |
|  | Model 7 | | | | | |
| normal | 0.959 | 0.904 | 0.936 | 0.938 | 0.921 | 0.983 |
| osteopenia | 0.807 | 0.940 | 0.836 | 0.928 | 0.866 | 0.940 |
| osteoporosis | 0.852 | 0.983 | 0.885 | 0.978 | 0.944 | 0.978 |
| Model 8 | | | | | | |
| normal | 0.920 | 0.970 | 0.978 | 0.890 | 0.935 | 0.992 |
| osteopenia | 0.922 | 0.923 | 0.823 | 0.968 | 0.904 | 0.973 |
| osteoporosis | 0.941 | 0.989 | 0.923 | 0.992 | 0.968 | 0.989 |
| Model 8 external validation | | | | | | |
| normal | 0.879 | 0.983 | 0.966 | 0.938 | 0.946 | 0.986 |
| osteopenia | 0.873 | 0.898 | 0.802 | 0.937 | 0.890 | 0.930 |
| osteoporosis | 0.918 | 0.956 | 0.910 | 0.960 | 0.943 | 0.975 |

**Note:** AUC-area under the ROC curve; 95%CI-95 % confidence interval; Se-sensitivity; Sp- specificity; PPV- positive predictive value; NPV- negative predictive value; Ac- accuracy.

**Appendix Table S5.** **Comparison of the efficacy of different models on the testing datasets**

|  | normal | *Z* | *P* | osteopenia | *Z* | *P* | osteoporosis | *Z* | *P* |
| --- | --- | --- | --- | --- | --- | --- | --- | --- | --- |
| Model 1 | 0.991 | 0.225 | 0.822 | 0.966 | 0.974 | 0.330 | **0.993** | 0.923 | **0.356** |
| Model 2 | **0.995** | 1.703 | **0.089** | 0.951 | 2.897 | 0.004 | **0.993** | 1.318 | **0.187** |
| Model 3 | 0.991 | 0.207 | 0.836 | 0.961 | 1.129 | 0.259 | 0.987 | 1.235 | 0.217 |
| Model 4 | 0.993 | 1.703 | 0.089 | 0.951 | 2.897 | 0.004 | 0.988 | 1.318 | 0.187 |
| Model 5 | 0.986 | 1.688 | 0.091 | 0.948 | 3.071 | 0.002 | 0.986 | 1.142 | 0.254 |
| Model 6 | 0.983 | 2.248 | 0.025 | 0.940 | 3.245 | 0.001 | 0.978 | 2.045 | 0.041 |
| Model 7 | 0.990 | 0.924 | 0.356 | 0.970 | 0.623 | 0.533 | 0.989 | 0.046 | 0.963 |
| Model 8 | 0.992 |  |  | **0.973** |  |  | 0.989 |  |  |

**Note:** Bold show the highest value of each performance indicator.
