# Supplementary material for: Feasibility study of opportunistic osteoporosis screening on chest CT using a multi-feature fusion DCNN model
Source: Arch Osteoporos. 2024 Oct 17;19(1):98. doi: 10.1007/s11657-024-01455-7 (PMC11485148; doi:10.1007/s11657-024-01455-7)
Supplement: Supplementary file 2 — Supplementary file2 (PDF 793 KB) [file 11657_2024_1455_MOESM2_ESM.pdf]

Supplementary document/ Appendix

Appendix Table S1. Details of CT protocols

CT information		Parameters	
		**Internal scanner**	**External scanner**
**CT system**		Ingenuity Core 128 CT	SOMATOM Force CT
**CT scan**	Tube voltage	120kVp	120kVp
	Time–current product	iPatient range: 100~300 mAs	CARE Dose4D
	Detector collimation	64×0.625mm	192×0.6mm
**CT image**	Image matrix	512×512	512×512
	Field of view	500×500mm	500×500mm
	Reconstruction section thickness	2.0mm	2.0mm

Appendix Table S2. Bone-ClassNet structure diagram

Layer Name	Output Size	Architecture
Conv 1	112×112	7×7,64, stride 2
Conv 2_x	56×56	3×3, maxpool, stride 2
		$\begin{bmatrix} 1\times1,64 \\ 3\times3,64 \\ 1\times1,256 \end{bmatrix}\times1 + \begin{bmatrix} 1\times1,64 \\ 3\times3,64 \\ 1\times1,256 \end{bmatrix}\times2$
		1×1,64, stride 1
Conv 3_x	28×28	$\begin{bmatrix} 1\times1,128 \\ 3\times3,128 \\ 1\times1,512 \end{bmatrix}\times1 + \begin{bmatrix} 1\times1,128 \\ 3\times3,128 \\ 1\times1,512 \end{bmatrix}\times3$
		1×1,512, stride 1
Conv 4_x	14×14	$\begin{bmatrix} 1\times1,256 \\ 3\times3,256 \\ 1\times1,1024 \end{bmatrix}\times1 + \begin{bmatrix} 1\times1,256 \\ 3\times3,256 \\ 1\times1,1024 \end{bmatrix}\times22$
		1×1,1024, stride 1
Conv 5_x	7×7	$\begin{bmatrix} 1\times1,512 \\ 3\times3,512 \\ 1\times1,2048 \end{bmatrix}\times1 + \begin{bmatrix} 1\times1,512 \\ 3\times3,512 \\ 1\times1,2048 \end{bmatrix}\times2$
		1×1, 2048, stride 1
	1×1	Average pool,2048-d fc, softmax

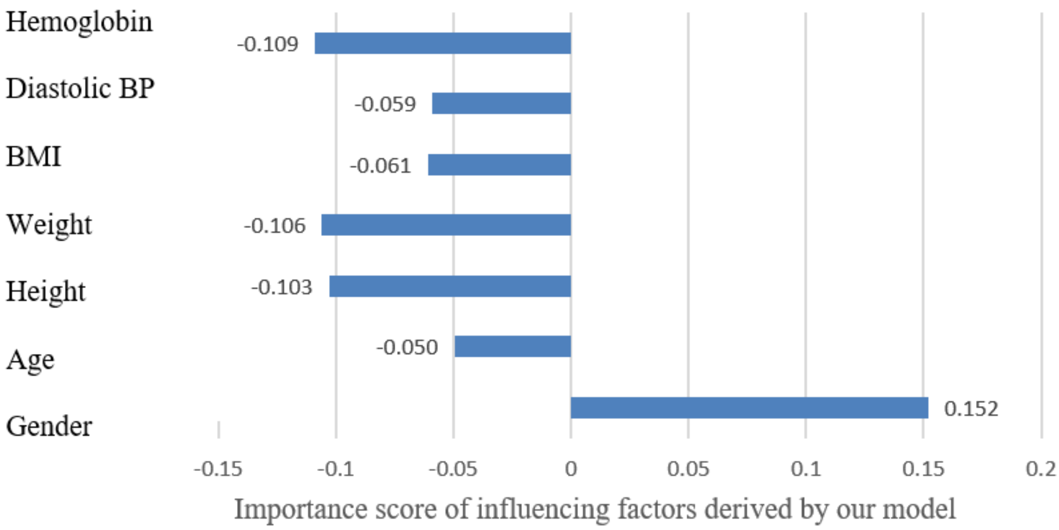

Appendix Fig. S1. 7 Influencing factors of Osteoporosis.

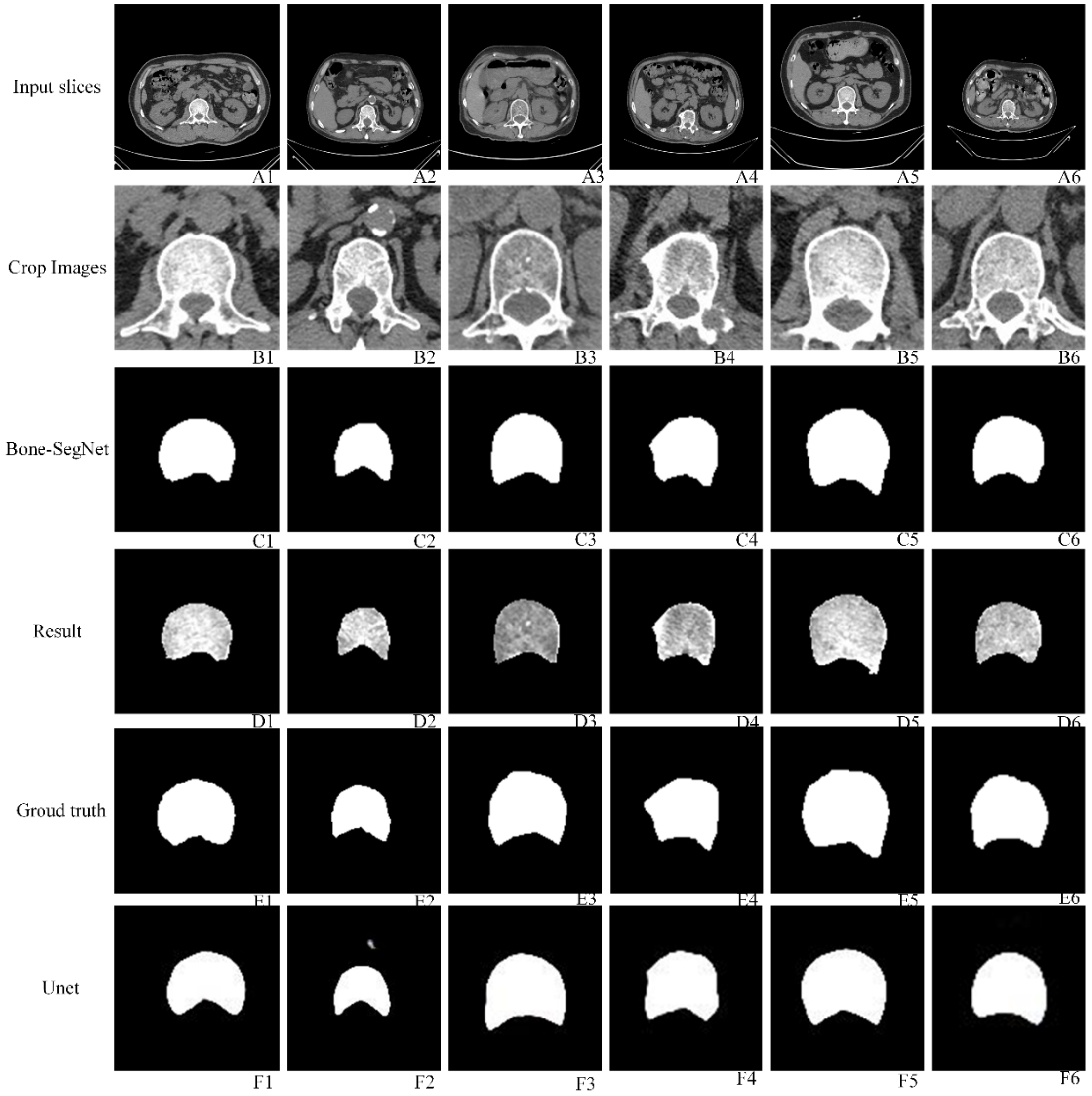

Appendix Fig.S2. Partial segmentation results. Fig. A1-F1: The female was 53 years old, who was diagnosed as normal bone mass with BMD $_{individual}$=140.7mg/cm^3. Fig. A2-F2: The male was 81

years old, who was diagnosed as normal bone mass with BMD $_{individual}$=133.8mg/cm^3. Fig. A3-F3: The female was 64 years old, who was diagnosed as osteoporosis with BMD $_{individual}$=54.3mg/cm^3. Fig. A4-F4: The male was 67 years old, who was diagnosed as osteoporosis with BMD $_{individual}$= 77.5 mg/cm^3. Fig. A5-F5: The male was 50 years old, who was diagnosed as osteopenia with BMD $_{individual}$=116.4 mg/cm^3. Fig. A6-F6: The male was 73 years old, who was diagnosed as osteopenia with BMD $_{individual}$= 102 mg/cm^3. Fig. A1-A6: The axial CT images of the central slice of lumbar 1 vertebral body of 6 subjects successively. Fig.B1-B6: Local magnification of the vertebral body in order to see the effect of the network; Fig. C1-C6: Bone-SegNet segmented images. Fig. D1-D6: Extracted ROI. Fig. E1-E6: Groud truth images. Fig. F1-F6: U-net segmented image.

Appendix Table S3. Introduction of eight classification models

Model name	Model type	Model description	Data source
Model 1	radiomics model, based on random forest classifier	ML model (nonlinear tree-based model) integrated learning method	L1 + L2
Model 2	radiomics model, based on naive Bayes	ML model based on Bayes theorem and independent assumption of feature conditions	L1 + L2
Model 3	DCNN model, based on Bone-ClassNet	convolutional neural network (CNN)	L1 + L2
Model 4	fusion model, radiomics features + DL	filtered omics features were fused with the DL features extracted by CNN-Net, and the full connection layer was used for classification	L1 + L2
Model 5	multi-feature fusion model clinical information + DL	DL features extracted from CNN-Net and clinical information were fused, and the full connectivity layer was used for classification	L1 + L2
Model 6	multi-feature fusion model clinical information + radiomics features + DL	filtered omics features, the DL features extracted by CNN-Net and clinical information were fused, and the full connectivity layer was used for classification	L1 + L2
Model 7	DCNN model, based on Bone-ClassNet	extracts DL features through CNN, uses the full connection layer for classification	L1*
Model 8	multi-feature fusion model clinical information + radiomics features + DL	integrates the filtered omics features, the DL features extracted by CNN-Net and clinical information, and uses the full connectivity layer for classification	L1*

Appendix Table S4. Effectiveness evaluation of different models on the testing datasets

categories	Se	Sp	PPV	NPV	Ac	AUC
Model 1						
normal	0.932	0.982	0.987	0.906	0.952	0.991
osteopenia	0.862	0.934	0.833	0.946	0.914	0.966
osteoporosis	0.941	0.964	0.787	0.991	0.962	0.993
Model 2						
normal	0.876	0.994	0.995	0.842	0.923	0.995
osteopenia	0.853	0.891	0.750	0.941	0.880	0.951
osteoporosis	0.961	0.956	0.753	0.994	0.956	0.993
Model 3						
normal	0.900	0.982	0.986	0.868	0.933	0.991
osteopenia	0.905	0.891	0.760	0.961	0.894	0.968
osteoporosis	0.843	0.978	0.843	0.978	0.962	0.987
Model 4						
normal	0.920	0.964	0.975	0.889	0.938	0.993
osteopenia	0.905	0.927	0.826	0.962	0.899	0.951
osteoporosis	0.961	0.986	0.907	0.995	0.962	0.988
Model 5						
normal	0.916	0.988	0.991	0.887	0.916	0.986
osteopenia	0.922	0.921	0.817	0.967	0.883	0.948
osteoporosis	0.941	0.980	0.873	0.992	0.966	0.986
Model 6						
normal	0.924	0.976	0.983	0.896	0.938	0.990
osteopenia	0.931	0.934	0.844	0.972	0.914	0.970
osteoporosis	0.980	0.989	0.926	0.997	0.976	0.989
Model 7						
normal	0.959	0.904	0.936	0.938	0.921	0.983
osteopenia	0.807	0.940	0.836	0.928	0.866	0.940
osteoporosis	0.852	0.983	0.885	0.978	0.944	0.978
Model 8						
normal	0.920	0.970	0.978	0.890	0.935	0.992
osteopenia	0.922	0.923	0.823	0.968	0.904	0.973
osteoporosis	0.941	0.989	0.923	0.992	0.968	0.989
Model 8 external validation						
normal	0.879	0.983	0.966	0.938	0.946	0.986
osteopenia	0.873	0.898	0.802	0.937	0.890	0.930
osteoporosis	0.918	0.956	0.910	0.960	0.943	0.975

Note: AUC-area under the ROC curve; 95%CI-95 % confidence interval; Se-sensitivity; Sp-specificity; PPV- positive predictive value; NPV- negative predictive value; Ac- accuracy.

Appendix Table S5. Comparison of the efficacy of different models on the testing datasets

	normal	*Z*	*P*	osteopenia	*Z*	*P*	osteoporosis	*Z*	*P*
Model 1	0.991	0.225	0.822	0.966	0.974	0.330	**0.993**	0.923	**0.356**
Model 2	**0.995**	1.703	**0.089**	0.951	2.897	0.004	**0.993**	1.318	**0.187**
Model 3	0.991	0.207	0.836	0.961	1.129	0.259	0.987	1.235	0.217
Model 4	0.993	1.703	0.089	0.951	2.897	0.004	0.988	1.318	0.187
Model 5	0.986	1.688	0.091	0.948	3.071	0.002	0.986	1.142	0.254
Model 6	0.983	2.248	0.025	0.940	3.245	0.001	0.978	2.045	0.041
Model 7	0.990	0.924	0.356	0.970	0.623	0.533	0.989	0.046	0.963
Model 8	0.992			**0.973**			0.989		

Note: Bold show the highest value of each performance indicator.
